# Benefits and risks of using laparoscopic ultrasonography versus intraoperative cholangiography during laparoscopic cholecystectomy for gallstone disease: a systematic review and meta-analysis

**DOI:** 10.1007/s00464-024-10979-5

**Published:** 2024-07-17

**Authors:** Anders Edebo, John Andersson, Joss Gustavsson, Lennart Jivegård, Darius Ribokas, Therese Svanberg, Susanna M. Wallerstedt

**Affiliations:** 1 Patient Safety and Quality Improvement, Sahlgrenska University Hospital, Gothenburg, Sweden; 2 Department of Surgery and Orthopedics, Hospitals in the West/Alingsås Hospital, Alingsås, Sweden; 3 Medical Library, Region Västra Götaland, Sahlgrenska University Hospital, Gothenburg, Sweden; 4 HTA-Centrum, Sahlgrenska University Hospital, Region Västra Götaland, Gothenburg, Sweden; 5 Department of Surgery and Orthopedics, Hospitals in the West /Högsbo NS Hospital, Gothenburg, Sweden; 6 Department of Pharmacology, Sahlgrenska Academy, University of Gothenburg, Box 431, 405 30 Gothenburg, Sweden

**Keywords:** Cholangiography, Cholecystectomy, Laparoscopic, Meta-analysis, Ultrasonography

## Abstract

**Background:**

Intraoperative laparoscopic ultrasonography (LUS) or intraoperative cholangiography (IOC) can be used for visualisation of the biliary tract during laparoscopic cholecystectomy. The aim of this systematic review was to compare use of LUS with IOC.

**Methods:**

PubMed, Embase, the Cochrane Library, and Web of Science were searched (last update: April 2024). PICO: P = patients undergoing intraoperative imaging of the biliary tree during laparoscopic cholecystectomy for gallstone disease; I = intervention: LUS; C = comparison: IOC; O = outcomes: mortality, bile duct injury, retained gallstone, conversion to open cholecystectomy, procedural failure, operation time including imaging time. Included articles were critically appraised using checklists. Conclusions were based on studies without major risk of bias. Meta-analyses were performed using random effects models. Certainty of evidence was assessed according to GRADE.

**Results:**

Sixteen non-randomised studies met the PICO. Two before/after studies (594 versus 807 patients) contributed to conclusions regarding mortality (no events; very low certainty evidence), bile duct injury (1 versus 0 events; very low certainty evidence), retained gallstone (2 versus 2 events; very low certainty evidence), and conversion to open cholecystectomy (6 versus 21 events; risk ratio: 0.38 (95% confidence interval: 0.15–0.95); I^2^ = 0%; low certainty evidence). Seven additional studies, using intra-individual comparisons, contributed to conclusions regarding procedural failure; risk ratio: 1.12 (95% confidence interval: 0.70–1.78; I^2^ = 83%; very low certainty evidence). No studies reported operation time. Mean imaging time for LUS and IOC, reported in 12 studies, was 4.8‒10.2 versus 10.9‒17.9 min (mean difference: − 7.8 min (95% confidence interval: − 9.3 to − 6.3); I^2^ = 95%; moderate certainty evidence).

**Conclusion:**

It is uncertain whether there is any difference in mortality/bile duct injury/retained gallstone using LUS compared with IOC, but LUS may be associated with fewer conversions to open cholecystectomy and is probably associated with shorter imaging time.

**Graphical Abstract:**

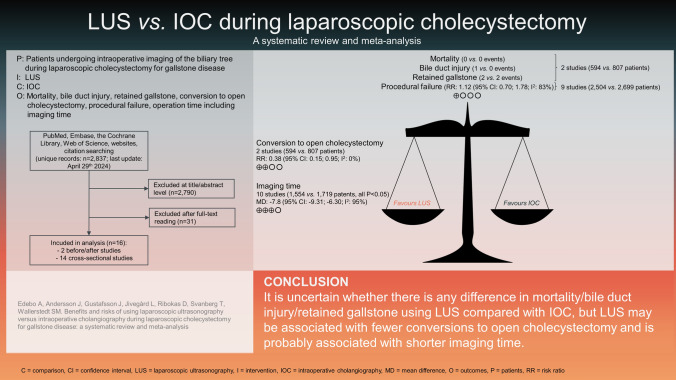

**Supplementary Information:**

The online version contains supplementary material available at 10.1007/s00464-024-10979-5.

In symptomatic gallstone disease, the established treatment is to remove the gallbladder, nowadays preferably by laparoscopic cholecystectomy. Intraoperative cholangiography (IOC) is the most frequently used method during cholecystectomy to visualise the biliary anatomy and to ensure that there are no residual stones in the biliary tract. Indeed, residual stones postoperatively imply a risk of developing jaundice, pancreatitis, and sepsis. IOC is also useful for intraoperative discovery of bile duct injuries, a dreaded complication that occurs in about 0.5% of the surgeries [1]; long-term bile duct injury-related mortality has been reported to range between 1.8% and 4.6%, and such injuries may also contribute to impaired quality of life [2].

Compared with IOC, laparoscopic ultrasonography (LUS) is a less invasive procedure for visualisation of the bile ducts; access to the biliary tract or injection of contrast medium is normally not needed. In addition, LUS has the advantage of providing an overview of adjacent anatomical structures, for instance, the cystic and hepatic arteries. In severe cholecystitis, this may be of particular value [3]. Other advantages of LUS include that visualisation can easily be repeated throughout the procedure and it does not involve ionising radiation. As with all forms of ultrasound examinations, however, a disadvantage of LUS is that it is user-dependent and requires training. Meta-analyses show that the sensitivity and the specificity for the detection of gallstones in the biliary tract by LUS is comparable to that of IOC [4, 5]. As far as we are aware, patient effects of using LUS compared with IOC have not previously been reviewed. This systematic review was undertaken to shed light on this issue.

## Methods

This systematic review was primarily conducted within a health technology assessment (HTA), intended to provide information for regional evidence-based decision-making and also including, for instance, the number of patients at issue in the region as well as economic aspects [6]. In the present study, we report the systematic review based on an updated literature search.

Our systematic review was performed according to the established routines at the regional HTA centre in Region Västra Götaland, Sweden, reported according to the Preferred Reporting for Systematic Reviews and Meta-Analyses (PRISMA) guidelines [7], and preregistered with the International Prospective Register of Systematic Reviews (PROSPERO; registration number: CRD42023404758).

The aim was defined in the PICO format. Accordingly, participants (P) were patients undergoing intraoperative imaging of the biliary tree during laparoscopic cholecystectomy because of gallstone disease. The intervention (I) was LUS, and the comparison (C) was IOC. Outcomes (O) were mortality, bile duct injury, retained gallstone, conversion to open cholecystectomy, procedural failure, and operation time including imaging time. Publications were restricted to randomised controlled trials (RCTs) and non-randomised controlled studies with ≥ 100 patients in each comparison group. Languages were restricted to English, Swedish, Danish, or Norwegian.

### Literature search and study selection

Two authors (J.G., T.S.) performed systematic searches in PubMed, Embase, the Cochrane Library and Web of Science (December 15th, 2022), with a subsequent update in all databases (April 29th, 2024). Reference lists of relevant articles were scrutinised for additional references. The websites of the Swedish Agency for Health Technology Assessment and Assessment of Social Services (SBU) and other local HTA centres were visited. To identify ongoing studies, a search in Clinicaltrials.gov was performed (March 20th, 2023). Search strategies are provided in the supplemental material.

Two authors independently screened titles and abstracts to exclude publications that clearly did not meet the PICO criteria. Abstracts obtained in the first systematic search were screened using the Rayyan tool [8]. Discrepancies were resolved in consensus. For the remaining publications, the full texts were retrieved and were independently assessed by at least two authors, after which all authors in the first systematic search, and two authors in the search update, in consensus finally decided on inclusion or exclusion according to PICO. For articles excluded in consensus, after full-text reading, reasons for exclusion were recorded.

### Data extraction and study assessments

For included studies, data on design and methodology were extracted, as well as data regarding participant and intervention characteristics. Number of events or measures of effect were also extracted. Data were independently extracted by two authors, with discrepancies resolved in consensus. We did not request additional data from the study investigators. The outcome procedural failure was defined as the opposite to procedural success, i.e., (i) that the imaging technique could not be applied, or (ii) that the biliary tree could not be visualised from confluence of the hepatic ducts to the pancreatic ampulla. Conversions to open surgery were not included in the analysis of procedural failure.

At least two authors independently appraised the included studies using checklists used by HTA-centrum, Sahlgrenska University Hospital, modified from checklists developed by the Swedish Agency for Health Technology Assessment and Assessment of Social Services [9]. Consensus discussions were then performed to decide on the domains directness, study limitations (risk of bias), and precision, in the categories + (plus; no or minor problems), ? (question mark; some problems), and – (minus; major problems). The assessments were performed at the outcome level. As decided beforehand, studies without major risk of bias formed the basis for the conclusions. The certainty of evidence was assessed using the Grading of Recommendations Assessment, Development and Evaluation (GRADE) approach [10].

### Statistical analysis

When two or more studies provided poolable data, we performed random effects meta-analyses using the software Review Manager (RevMan) version 5.4.1 (The Nordic Cochrane Centre, The Cochrane Collaboration, Copenhagen, Denmark) to obtain the risk ratio (RR), the risk difference, and the mean difference, along with the 95% confidence interval (CI). Regarding the outcome procedural failure, we required studies to report both application and visualisation failures to be included in the meta-analysis; otherwise, they were considered to have major study limitations regarding this outcome. Studies with inter- and intra-individual comparisons were considered adequate to pool regarding the outcomes procedural failure and operation time including imaging time.

## Results

**Fig. 1:**
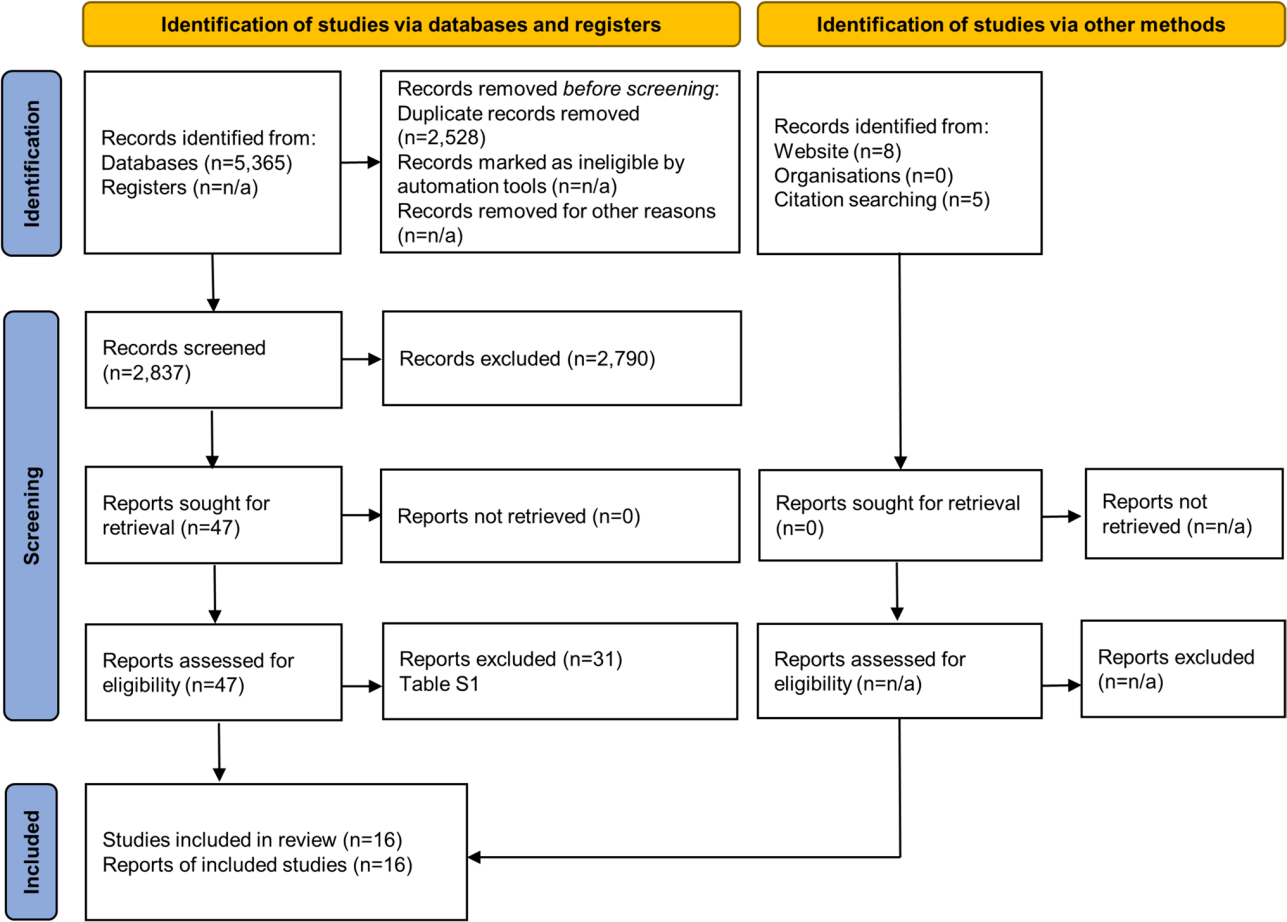
PRISMA flowchart

After removal of duplicates, the literature search identified 2837 unique publications, 16 of which were included in this systematic review (Fig. 1) [11–26]. Publications excluded after full-text reading, as well as the reasons for excluding them, are presented in Table S1.

Included studies are described in Table 1 and assessments underlying directness and risk of bias assessments clarified in Table S2; precision was considered in the GRADE process. Some of the studies included inter-individual comparisons [15, 16, 19, 26], whereas others reported intra-individual comparisons only [11–14, 17, 18, 20–25]. The studies were performed in the United States [11, 15, 18, 19, 22–26], France [13, 14], Switzerland [20, 21], Germany [12], Belgium [16], and China [17]. In several studies, one single surgeon performed LUS [15, 16, 19, 21, 24–26], one study included seven LUS-performing surgeons [13] and one study included three sites [23]. In the remaining studies, the number of LUS-performing surgeons was not reported [11, 12, 14, 17, 18, 20, 22]. LUS experience was reported in five studies, with five to 100 previous procedures required before data collection [13, 15, 21, 23, 25].

**Table 1:** Characteristics of the included studies

Study Year	Country	Design	Patients				Intervention		
			Indication^a^	n	Age	Female sex	Performing physician(s) n	LUS experience	LUS frequency (MHz)
Barteau et al*.* 1995 [10]	USA	Cross-sectional (intra-individual comparison)	All indications	125	mean ± SEM: 54.6 ± 1.4	73%	NR	NR	7.0/10
Birth et al*.* 1998 [11]	Germany	Cross-sectional (intra-individual comparison)	Elective cholecystitis	518	mean (range): 51.7 (15‒88)	75%	NR	NR	7.5
Catheline et al*.* 1999 [12]	France	Cross-sectional (intra-individual comparison)	All indications	600	mean (range): 54 (21‒94)	72%	7	5 previous procedures required	5/6.5/7.5
Catheline et al*.* 2002 [13]	France	Cross-sectional (intra-individual comparison)	All indications	900	mean (range): 52 (18‒95)	71%	NR	NR	NR
Halpin et al*.* 2002 [14]	USA	Before/after	All indications	I: 394 C: 400	mean ± SEM: I: 48.1 ± 0.7 C: 48.8 ± 0.8	I: 78% C: 75%	1	Transition period: first 100 LUS excluded	7.5
Hublet et al*.* 2009 [15]	Belgium	Cross-sectional (inter-individual comparison)	All indications	I: 269 C: 695	NR	NR	1	NR	7.5
Li et al*.* 2009 [16]	China	Cross-sectional (intra-individual comparison)	Score system to include those who might have occult choledocholithiasis, and to exclude those who were preferred to undergo ERCP before laparoscopic cholecystectomy	103	mean (range): 48.7 (25‒85)	63%	NR	NR	NR
Machi et al*.* 1999 [17]	USA	Cross-sectional (intra-individual comparison)	All indications	100	mean: 55.9 (SD/SEM/range NR)	71%	NR	NR	5/7/8
Perry et al*.* 2008 [18]	USA	Cross-sectional (inter/intra-individual comparisons)	Cholelithiasis	I: 236 C: 239 (partly overlapping; 104 had both)	NR	NR	1	NR	5‒10
Röthlin 1996 [19]	Switzerland	Cross-sectional (intra-individual comparison)	All indications	158	mean (range): 52 (19‒80)	63%	NR	NR	5.5/5‒7.5
Röthlin et al*.*, 1996 [20]	Switzerland	Cross-sectional (intra-individual comparison)	All indications	200 Cohort I: 100 Cohort II: 100	mean (range): Cohort I: 52.0 (19‒80) Cohort II: 49.8 (17‒81)	Cohort I:60% Cohort II: 73%	1	30 LUS performed prior to this study	5.5/5‒7.5
Siperstein et al*.* 1999 [21]	USA	Cross-sectional (intra-individual comparison)	All indications	300	NR	NR	NR	NR	NR
Stiegmann et al*.* 1995 [22]	USA	Cross-sectional (intra-individual comparison)	Elective or semi-elective laparoscopic cholecystectomy	209	mean: 46 (SD/range NR)	78%	NR, 3 sites	≥ 20 LUS	7.5
Thompson et al*.* 1998 [23]	USA	Cross-sectional (intra-individual comparison)	All indications	140	mean (range): 54.9 (22‒93)	71%	1	NR	7
Tranter et al*.* 2003 [24]	USA	Cross-sectional (intra-individual comparison)	All indications	135	mean: 53 (SD/range NR)	77%	1	80 LUS performed prior to this study	7.5
Wu et al*.* 1998 [25]	USA	Before/after	All indications	I: 200 C: 407	mean ± SEM: I: 49 ± 1 C: 49 ± 1	I: 74% C: 74%	1	NR	7.5

*C* comparison, *ERCP* endoscopic retrograde cholangiopancreatography, *I* intervention, *LUS* laparoscopic ultrasonography, *NA* not applicable, *NR* not reported

^a^Within laparoscopic cholecystectomy for gallstone disease

Conclusions on mortality, bile duct injuries, and retained gallstone were based on two before/after studies including 594 versus 807 patients in the intervention and comparison groups, respectively (Table 2) [15, 26]. Both studies had a follow-up of one month. In the GRADE process, starting from low certainty evidence as only non-randomised studies were available [10], we downgraded two steps because of very serious imprecision; the studies contained no or few events. In conclusion, it is uncertain whether there is any difference in the risk of mortality, bile duct injuries, and retained gallstone using LUS compared with IOC (GRADE ).

**Table 2:** Study results, LUS versus IOC, and assessments of directness and risk of bias

Author Year	Mortality	Bile duct injury	Retained gallstone	Conversion to open cholecystectomy	Procedural failure^a^	Imaging time^c^ (minutes)	Assessment^d^	
							Directness	Risk of bias
Barteau et al*.* 1995 [10]	NA	NA	NA	NA	14/125 *vs.* 2/125 Application failure: 3 *vs.*2 Visualisation failure: 11 *vs.* 0	mean ± SEM 6.6 ± 0.3 *vs.* 10.9 ± 0.6	?	?
Birth et al*.* 1998 [11]	NA	NA	NA	NA	79^b^/518 *vs.* 46^b^/518 Application failure: 2 *vs.* 41 Visualisation failure: 77^a^ *vs.* 5^a^	mean (range) 7 (3‒25) *vs.* 16 (5‒45)	?	+
Catheline et al*.* 1999 [12]	NA	NA	NA	NA	78^b^/600 *vs.* 127^b^/600 Application failure: 0 *vs.* 102 Visualisation failure: 78 *vs.*25	mean (range) 10.2 (5‒20) *vs.* 17.9 (7‒38)	?/ +	?
Catheline et al*.* 2002 [13]	NA	NA	NA	NA	0 + ?/900 *vs.* 138 + ?/900 Application failure: 0 *vs.* 138 Visualisation failure: NR	mean (range) 9.8 (4‒21) *vs.* 17.6 (7‒42)	+	–/ +
Halpin et al*.* 2002 [14]	0/380 *vs.* 0/374	1/380 *vs.* 0/374	1/380 *vs.*1/374	4/394 *vs.* 11/400	47/390 *vs.* 38/388 Application failure:11 *vs.* 26 Visualisation failure: 36 *vs.* 12	mean ± SEM 5.1 ± 0.1 *vs.* 16.0 ± 0.5	?	?
Hublet et al*.* 2009 [15]	NR	0/271 *vs.* 5/730	0/271 *vs.*1/730	NR	2/271 *vs.* 35 + ?/730 Application failure:0 *vs.* 35 Visualisation failure: 2 *vs.* NR	NR	?	–
Li et al*.* 2009 [16]	NA	NA	NA	NA	27^b^/103 *vs.* 122/103 Application failure: 0 *vs.* 9 Visualisation failure: 27 *vs.* 3	mean ± SD 8.5 ± 1.9 *vs.* 13.8 ± 3.7	–	–
Machi et al*.* 1999 [17]	NA	NA	NA	NA	5/100 *vs.* 8/100 Application failure: 0 *vs.* 6 Visualisation failure: 5 *vs.* 2	mean ± SD 8.2 ± 3.5 15.9 ± 6.8	?	?
Perry et al*.* 2008 [18]	NR	0/132 *vs. *0/135	NR	NR	11/236 *vs.* 11/239 Application failure: 3 *vs.* 11 Visualisation failure: 8 *vs.* 0	NR	?	–
Röthlin 1996 [19]	NA	NA	NA	NA	0 + ?/158 *vs.* 12 + ?/158 Application failure: 0 *vs.* 17 Visualisation failure: NR	mean ± SD 4.8 ± 1.9 14.1 ± 4.1	?	–/?
Röthlin et al*.*, 1996 [20]	NA	NA	NA	NA	0 + ?/200 *vs.* 17 + ?/200 Application failure: 0 *vs.* 17 Visualisation failure: NR	mean ± SD Cohort I: 5.4 ± 2.4 *vs.* 16.4 ± 7.4 Cohort II: 4.5 ± 1.7 *vs.* 13.5 ± 4.5	?	–/?
Siperstein et al*.* 1999 [21]	NA	NA	NA	NA	0 + ?/300 *vs.* 18 + ?/300 Application failure: 0 *vs.* 18 Visualisation failure: NR	NR	?	–
Stiegmann et al*.* 1995 [22]	NA	NA	NA	NA	24/202 *vs.* 15/201 Application failure: NR Visualisation failure: NR	mean ± SD 7.0 ± 3.3 *vs.*13.4 ± 5.7	+	?
Thompson et al*.* 1998 [23]	NA	NA	NA	NA	NR	mean ± SEM 6.6 ± 0.3 *vs.*10.9 ± 0.6	?	?
Tranter et al*.* 2003 [24]	NA	NA	NA	NA	3/135 *vs.* 14/135 Application failure: 2 *vs.* 9 Visualisation failure: 1 *vs.* 5	NR	?	?
Wu et al*.* 1998 [25]	0/172 *vs.* 0/381	0/172 *vs. *0/381	1/172 *vs.*1/381	2/200 *vs.*10/407	14/198 *vs.* 23/393 Application failure: 14 *vs.* 12 Visualisation failure: 0 *vs.* 11	mean ± SEM 5.3 ± 0.2 *vs.* 15.1 ± 0.4	?	?/ +

*IOC* intraoperative cholangiography, *LUS* laparoscopic ultrasonography, *NA* not applicable, *NR* not reported, *SD* standard deviation, *SEM* standard error of the mean

^a^Procedural failure was defined as the opposite to procedural success, i.e., (i) that the imaging technique could not be applied, or (ii) that the biliary tree could not be visualised from confluence of the hepatic ducts to the pancreatic ampulla

^b^Number of patients not summarised over the visualised bile duct segments, maximum number presented

^c^Operation time was not reported in any study

^d^ +  = no or minor problems, ? = some problems, – = major problems; aspects contributing to downgrading is described in Table S2

**Fig. 2:**
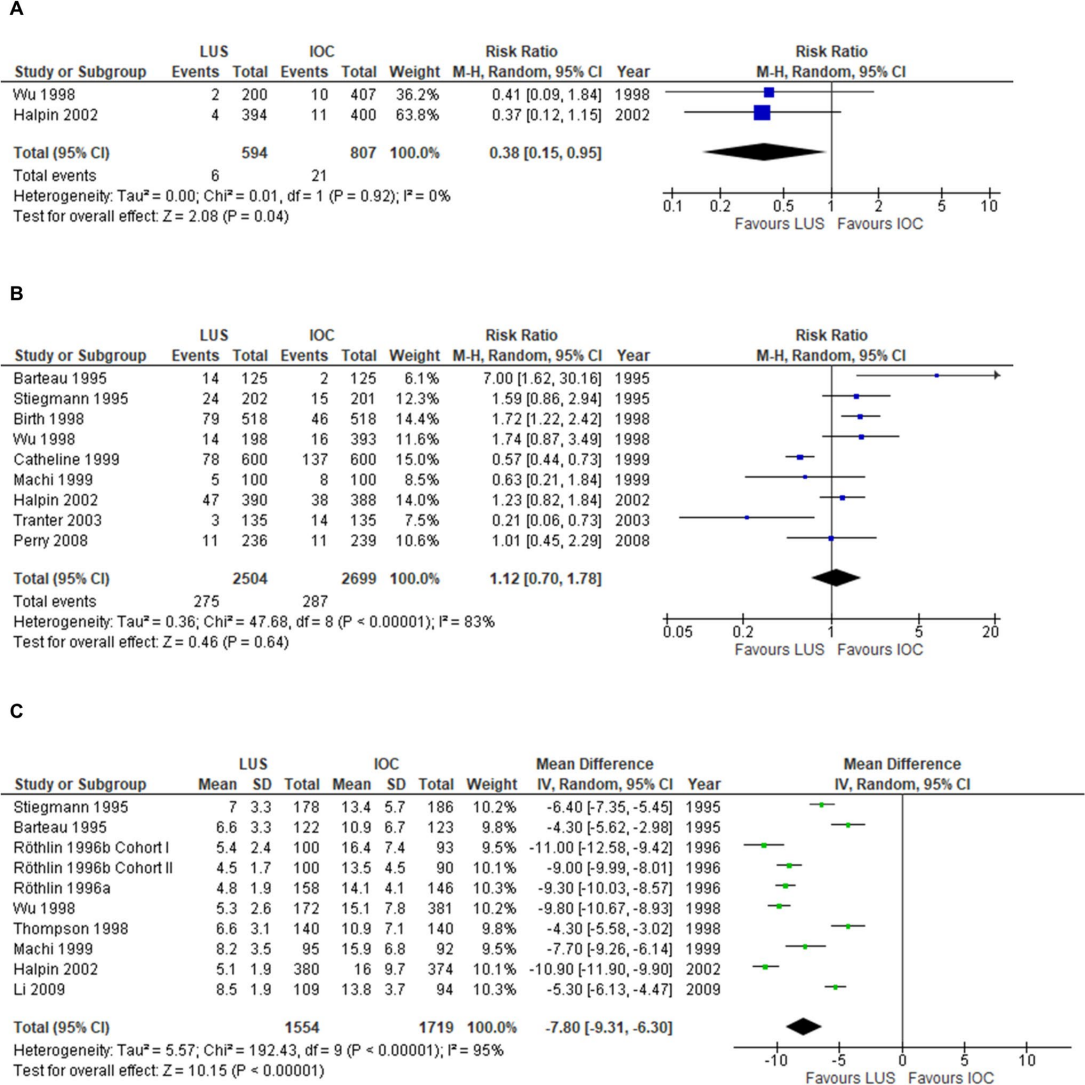
Forest plots and meta-analyses of studies without major risk of bias providing results regarding conversion to open cholecystectomy (**A**), procedural failure (**B**), and imaging time (**C**)

Regarding conversion to open cholecystectomy, the conclusion was also based on the two studies above (Table 2) [15, 26]. In all, 6 versus 21 events were reported, resulting in a pooled risk ratio of 0.38 (95% CI: 0.15–0.95; Fig. 2A), and a pooled risk difference of − 1.6 (95% CI: − 3.0 to − 0.2) percentage points. In the GRADE process, there was some uncertainty regarding directness as LUS is user-dependent and underlying evidence was restricted to single surgeon studies. Furthermore, there was uncertain precision as the confidence interval was wide. We did not consider these aspects sufficient for further downgrading. In conclusion, intraoperative LUS may be associated with fewer conversions to open surgery, compared with IOC (GRADE ).

Procedural failure was reported in 15 studies, four of which including inter-individual comparisons [15, 16, 19, 26] and eleven providing intra-individual comparisons only [11–14, 17, 18, 20–23, 25]. In nine studies without major risk of bias (3184 unique patients), 275 versus 287 events were reported in the intervention (*n* = 2504) and control (*n* = 2699) groups, resulting in a pooled risk ratio of 1.12 (95% CI: 0.70–1.78; Fig. 2B), and a pooled risk difference of 1 (95% CI: − 4 to 5) percentage points. Six studies reported application and visualisation failures separately [11, 15, 18, 19, 25, 26]. For LUS overall, the distribution between application and visualisation failures was 35% and 65%, respectively. For IOC, the distribution was the opposite; 69% were application failures and 31% visualisation failures. In the GRADE process, we downgraded two steps because of very serious inconsistency; four studies showed statistically significant results, two of which favouring LUS [11, 12] and two IOC [13, 25]. Furthermore, two of these studies explicitly included more than one surgeon [12, 13], also with statistically significant results in opposite directions. In conclusion, it is uncertain whether there is any difference in procedural failure using LUS compared with IOC (GRADE ).

No studies provided results regarding operation time. Imaging time was reported in 12 studies, two of which made inter-individual [15, 26], and ten intra-individual [11–14, 17, 18, 20, 21, 23, 24], comparisons. No studies were assessed to have major risk of bias regarding this outcome. In all, the studies included 3,854 patients. Mean imaging time for LUS ranged from 4.8 to 10.2 min, whereas mean imaging time for IOC ranged from 10.9 to 17.9 min. A meta-analysis, including studies that provided mean values, as well as standard deviation or standard error of the mean, revealed a pooled mean difference of − 7.8 min (95% CI: − 6.3 to − 9.3) (Fig. 2C). In the GRADE process regarding imaging time, a subset of the outcome operation time, there was no indirectness or inconsistency; all studies reported statistically significant reductions, between four and eleven minutes, and several surgeons/sites were involved in some studies. We upgraded one step because of a large magnitude of effect; dichotomisation of imaging time, e.g. ,< / ≥ 10 min, would likely result in a risk ratio < 0.5/ > 2 favouring LUS, i.e., the limit for upgrading [10]. In conclusion, no studies provide information regarding the operation time, but LUS is probably associated with shorter imaging time compared with IOC (GRADE  ).

The search in Clinicaltrials.gov resulted in 100 records, none of which fulfilled our PICO.

## Discussion

This systematic review shows that no conclusions can be drawn regarding potential effects of LUS versus IOC during laparoscopic cholecystectomy with regard to mortality, bile duct injury, retained gallstone, and procedural failure. LUS may, however, be associated with fewer conversions to open cholecystectomy and is probably associated with a shorter imaging time.

Regarding the possibly lower conversion rate for LUS, it may be speculated that an underlying factor could be that LUS allows visualisation of adjacent anatomical structures. This aspect may be particularly important in the presence of severe inflammation. Indeed, it has been argued that using LUS repeatedly during dissection, for visualisation of the biliary tract as well as the adjacent anatomical structures, could be particularly helpful during arduous surgery, for instance in case of acute or chronic cholecystitis, to avoid conversion to open cholecystectomy [27].

Operation time, the preferred time-related outcome from an overall perspective including both the imaging procedure per se and the surgical procedure, was not reported in any of the included studies. For imaging time, however, our evidence synthesis shows that LUS, on average, is associated with a 7.8-min shorter imaging time. In high-volume centres, such a procedure-related consequence, corresponding to almost 10% of the 83- to 89-min total surgery time [28], can be relevant, in particular if IOC is performed routinely and the operation theatre then can be used for an additional operation. In Sweden, IOC is undertaken or attempted in 86.4% of all cholecystectomies [29]. If imaging is only used in selective cases, on the other hand, provided that LUS expertise is available, the limited time required may lower the threshold for bile duct visualisation. Another method of interest for time-saving reasons is intraoperative fluorescence cholangiography (IFC), which has been associated with shorter operation time, but is also described to have limitations regarding the visualisation of gallstones [30, 31].

Interestingly, despite 15 non-randomised studies whereof nine were without major risk of bias, it is uncertain whether there are any differences between LUS and IOC regarding procedural failure. The wide range in results regarding this outcome, including the fact that two studies favoured LUS [11, 12] and two IOC [13, 25], may reflect the intrinsic difficulties associated with each procedure. LUS, with procedural failures ranging from 2 to 15%, has the limitation of being user-dependent, the ampulla being a particularly challenging area for visualisation. To facilitate interpretation, simultaneous infusion of sodium chloride could be useful, a procedure used in several studies, either into the bile ducts [24, 32] or into the right upper quadrant [3, 11, 15, 16, 19, 20, 23, 25, 26, 33, 34]. IOC, on the other hand, was more often subjected to application than visualisation failures. In this context, it may be of interest to note that compared with IOC, IFC has been reported favourable regarding visualisation of the common hepatic duct [35].

The ultrasonography technique has evolved over time. For instance, a high sonographic frequency can be applied to facilitate interpretation. Although our forest plot on procedural failure visually suggests a correlation between year of study and result, there was no obvious corresponding correlation with MHz applied. Indeed, most studies reporting this technical detail used 7.5 MHz [12, 15, 16, 23, 25, 26] and no study used more than 10 MHz, compared with presently available equipment where 12 MHz is often used for visualisation of the biliary tree. For IOC, it can be speculated that the wide range of procedural failures, from 1.6% to 23%, may reflect the difference between routine and selective use; IOC performed in selective cases may be associated with less experience, and thus more challenges in the cannulation step, in particular when the cystic duct is obliterated because of inflammation, fibrosis, or gallstone. Furthermore, during challenging IOC, cystic duct gallstones can be flushed into the common bile duct.

Although our systematic review identified 16 non-randomised studies that met the PICO, only four included inter-individual comparisons and only two of these, including 1,405 patients were without major risk of bias. In addition, LUS was performed by a single surgeon in these studies, an aspect that may be of importance for the generalisability of the results. The evidence base regarding the comparison LUS versus IOC in laparoscopic cholecystectomy can therefore be considered very restricted, in particular regarding rare patient events such as postoperative deaths, bile duct injuries, and retained gallstones.

In conclusion, as this systematic review shows that no conclusions can be drawn regarding LUS versus IOC with respect to critical patient outcomes and yet with promising results regarding conversions to open cholecystectomy and imaging time, further well-designed studies can be considered highly warranted.

### Supplementary Information

Below is the link to the electronic supplementary material.

Supplementary file1 (DOCX 79 KB)
