# Supplementary material for: Benefits and risks of using laparoscopic ultrasonography versus intraoperative cholangiography during laparoscopic cholecystectomy for gallstone disease: a systematic review and meta-analysis
Source: Surg Endosc. 2024 Jul 17;38(9):5096–107. doi: 10.1007/s00464-024-10979-5 (PMC11362195; doi:10.1007/s00464-024-10979-5)
Supplement: Supplementary file 1 — Supplementary file1 (DOCX 79 KB) [file 464_2024_10979_MOESM1_ESM.docx]

**Supplemental material**

Supplemental information to: Edebo A, Andersson J, Gustavsson J, Jivegård L, Ribokas D, Svanberg T, Wallerstedt SM. Benefits and risks of using laparoscopic ultrasonography versus intraoperative cholangiography during laparoscopic cholecystectomy for gallstone disease – a systematic review and meta-analysis

**Table of content**

| Search strategies | | Page 2 |
| --- | --- | --- |
| Table S1 | Studies excluded after full-text reading, and reasons for exclusion | Page 5 |
| Table S2 | Reasons underlying study assessments of directness and risk of bias | Page 6 |
| References | Full references: Included studies & Excluded studies | Page 8 |

**Search strategies**

**Database:** PubMed

**Date:** December 15^th^ 2022
**No. of results:** 1,702

**Search updated:** October 4^th^ 2023 and April 29^th^ 2024: 68 + 45 results

| **Search** | **Query** | **Items found** |
| --- | --- | --- |
| **#20** | **Search: #12 NOT #15 Filters: Danish, English, Norwegian, Swedish** | **1,702** |
| #16 | Search: #12 NOT #15 | 2,027 |
| #15 | Search: #13 OR #14 | 5,468,262 |
| #14 | Search: animal[ti] OR animals[ti] OR rat[ti] OR rats[ti] OR mouse[ti] OR mice[ti] OR rodent[ti] OR rodents[ti] OR dog[ti] OR dogs[ti] OR cat[ti] OR cats[ti] OR koalas[ti] OR hamster[ti] OR hamsters[ti] OR rabbit[ti] OR rabbits[ti] OR swine[ti] OR pigs[ti] OR murine[ti] OR porcine[ti] OR horses[ti] or horse[ti] | 2,118,272 |
| #13 | Search: animals[mh] NOT (animals[mh] AND humans[mh]) | 5,072,156 |
| #12 | Search: #10 NOT #11 | 2,044 |
| #11 | Search: block[ti] | 56,748 |
| [#10](https://www.ncbi.nlm.nih.gov/pubmed) | Search: #3 AND #6 AND #9 | 2,142 |
| [#9](https://www.ncbi.nlm.nih.gov/pubmed) | Search: #7 OR #8 | 517,953 |
| [#8](https://www.ncbi.nlm.nih.gov/pubmed) | Search: ultrasound[tiab] OR ultrasonography[tiab] | 381,085 |
| [#7](https://www.ncbi.nlm.nih.gov/pubmed) | Search: "Ultrasonography"[Mesh:NoExp] OR "Ultrasonography, Doppler"[Mesh] | 271,989 |
| [#6](https://www.ncbi.nlm.nih.gov/pubmed) | Search: #4 OR #5 | 166,138 |
| [#5](https://www.ncbi.nlm.nih.gov/pubmed) | Search: laparoscop*[tiab] | 148,083 |
| [#4](https://www.ncbi.nlm.nih.gov/pubmed) | Search: "Laparoscopy"[Mesh] | 114,158 |
| [#3](https://www.ncbi.nlm.nih.gov/pubmed) | Search: #1 OR #2 | [45,964](https://www.ncbi.nlm.nih.gov/pubmed/?cmd=HistorySearch&querykey=3) |
| [#2](https://www.ncbi.nlm.nih.gov/pubmed) | Search: cholecystectom*[tiab] | [32,803](https://www.ncbi.nlm.nih.gov/pubmed/?cmd=HistorySearch&querykey=2) |
| [#1](https://www.ncbi.nlm.nih.gov/pubmed) | Search: "Cholecystectomy"[Mesh] OR "Gallbladder/surgery"[Mesh] OR "Gallstones/surgery"[Mesh] | [34,742](https://www.ncbi.nlm.nih.gov/pubmed/?cmd=HistorySearch&querykey=1) |

**Database: Embase**1974 to 2022 December 14 (Ovid)

**Date:** December 15^th^ 2022

**No. of results:** 1,448
**Search updated:** April 29^th^ 2024: 16 results

| **#** | **Searches** | **Results** |
| --- | --- | --- |
| 1 | exp *cholecystectomy/ | 23,117 |
| 2 | exp *gallbladder/su [Surgery] | 307 |
| 3 | exp *gallstone/su [Surgery] | 2,542 |
| 4 | cholecystectom$.ab,kf,ti. | 45,907 |
| 5 | 1 or 2 or 3 or 4 | 49,673 |
| 6 | exp *laparoscopy/ | 85,571 |
| 7 | laparoscop$.ab,kf,ti. | 240,214 |
| 8 | 6 or 7 | 245,354 |
| 9 | *echography/ or exp doppler ultrasonography/ or peroperative echography/ | 145,625 |
| 10 | (ultrasound or ultrasonography).ab,kf,ti. | 585,040 |
| 11 | 9 or 10 | 649,970 |
| 12 | 5 and 8 and 11 | 2,811 |
| 13 | block.ti. | 60,641 |
| 14 | 12 not 13 | 2,678 |
| 15 | animal/ not (animal/ and human/) | 1,169,955 |
| 16 | (animal or animals or rat or rats or mouse or mice or rodent or rodents or dog or dogs or cat or cats or koalas or hamster or hamsters or rabbit or rabbits or swine or pigs or murine or porcine or horses or horse).ti. | 2,257,999 |
| 17 | 15 or 16 | 3,144,808 |
| 18 | 14 not 17 | 2,664 |
| 19 | limit 18 to (embase or medline) | 1,743 |
| **20** | **limit 19 to (danish or english or norwegian or swedish)** | **1,448** |

**Database:** The Cochrane Library
**Date:** December 15^th^ 2022

**No of results:** 100

*Cochrane Reviews (1)*

*Trials (99)*

**Search updated:** April 29^th^ 2024: *21 trials, 0 reviews*

| **ID** | **Search name:** | **Hits** |
| --- | --- | --- |
| #1 | MeSH descriptor: [Cholecystectomy] explode all trees | 2,059 |
| #2 | MeSH descriptor: [Gallbladder] explode all trees and with qualifier(s): [surgery - SU] | 35 |
| #3 | MeSH descriptor: [Gallstones] explode all trees and with qualifier(s): [surgery - SU] | 194 |
| #4 | (cholecystectom*):ti,ab,kw (Word variations have been searched) | 6,283 |
| #5 | #1 OR #2 OR #3 OR #4 | 6,355 |
| #6 | MeSH descriptor: [Laparoscopy] explode all trees | 6,594 |
| #7 | (laparoscop*):ti,ab,kw (Word variations have been searched) | 24,492 |
| #8 | #6 OR #7 | 24,613 |
| #9 | MeSH descriptor: [Ultrasonography] this term only | 5,140 |
| #10 | MeSH descriptor: [Ultrasonography, Doppler] explode all trees | 2,965 |
| #11 | (ultrasonography OR ultrasound):ti,ab,kw (Word variations have been searched) | 47,838 |
| #12 | #9 OR #10 OR #11 | 48,868 |
| #13 | #5 AND #8 AND #12 | 404 |
| #14 | (block):ti (Word variations have been searched) | 17,766 |
| #15 | #13 NOT #14 | 201 |
| #16 | (clinicaltrials OR trialsearch):so | 442,149 |
| #17 | #15 NOT #16 | 121 |
| #18 | (conference proceeding):pt | 214,308 |
| **#19** | **#17 NOT #18** | **100** |

**Database: Web of Science Core Collection**

**Date:** December 15^th^ 2022

**No. of results:** 1,747

**Search updated:** April 29^th^ 2024: 218 results

| **#** | **Search query** | **Results** |
| --- | --- | --- |
| 1 | cholecystectom* (Topic) | 33,528 |
| 2 | laparoscop* (Topic) | 170,412 |
| 3 | ultrasound OR ultrasonography (Topic) | 492,872 |
| 4 | #3 AND #2 AND #1 | 2,033 |
| 5 | TI=(block) | 198,616 |
| 6 | #4 NOT #5 | 1,900 |
| 7 | TI=(animal or animals or rat or rats or mouse or mice or rodent or rodents or dog or dogs or cat or cats or koalas or hamster or hamsters or rabbit or rabbits or swine or pigs or murine or porcine or horses or horse) | 2,708,340 |
| 8 | #6 NOT #7 | 1,878 |
| **9** | **#6 NOT #7 and English (Languages)** | **1,747** |

The websites of Statens beredning för medicinsk och social utvärdering (SBU), Folkehelseinstituttet, and Swedish regional HTA-agencies were visited in March 2023. Nothing directly answering the question at issue was found.

| **Sources** | **Search words [in Swedish]** | **Hits** | **Relevant hits** |
| --- | --- | --- | --- |
| **Swedish Agency for Health Technology Assessment and Assessment of Social Services [Statens beredning för medicinsk och social utvärdering, SBU]**  www.sbu.se | Gallsten  Ultraljud  Kolecystektomi | 8 | 0 |
| **Folkehelseinstituttet**  https://www.fhi.no/ku/metodevurdering/ | Browsed the category ”Metodevurdering” |  | 0 |
| **CAMTÖ**  https://www.regionorebrolan.se/sv/forskning/kontakt-och-organisation/hta-enheten-camto/ | Browsed |  | 0 |
| **HTA Region Stockholm**  https://www.chis.regionstockholm.se/hta/rapporter/ | Browsed |  | 0 |
| **Regional samverkansgrupp HTA (tidigare Metodrådet) i Sydöstra sjukvårdsregionen**  https://sydostrasjukvardsregionen.se/samverkansgrupper/hta/genomforda-bedomningar/ | Browsed |  | 0 |
| **HTA Syd**  https://vardgivare.skane.se/kompetens-utveckling/sakkunniggrupper/hta-skane/#110365 | Browsed |  | 0 |
| **Medicinska rådet, Region Dalarna**  https://www.regiondalarna.se/plus/vard/ovrig-halso--och-sjukvard/medicinska-radet/ | Browsed |  | 0 |

**Ongoing trials**

A search in Clinicaltrials.gov was performed on March 20^th^ 2023, using the search terms (cholecystectomy OR cholecystectomies) AND ((laparoscopic OR laparoscopy) AND (ultrasound OR ultrasonography) OR LUS)).

**Table S1** Studies excluded after full-text reading, as well as the reason for excluding them.

(For full citations, see pp 8‒9)

| **Author Year** | **Reason for exclusion** |
| --- | --- |
| Antal *et al.* 1994 | No comparison of I and C |
| Awan *et al.* 2023 | Wrong design: systematic review |
| Aziz *et al.* 2014 | Wrong design: meta-analysis |
| Bezzi *et al.* 1998 | Too few patients for comparison |
| Biffl *et al.* 2001 | Too few patients for comparison |
| Bush *et al.* 2022 | C: Too few patients (n=80) |
| Catheline *et al.* 1998 | Same patients as Catheline *et al.* 1999 |
| Chandra *et al.* 2017 | I/C missing: descriptive analysis of saline sono-cholangiography |
| Deziel 2022 | Wrong design: case series |
| Dili *et al.* 2017 | Wrong design: systematic review |
| Fisher *et al.* 2022 | Wrong design: systematic review |
| Hashimoto *et al.* 2010 | Wrong C: ultrasonography-guided IOC |
| Jakimowicz 1991 | No comparison of I and C |
| Jakimowicz 1993 | No comparison of I and C |
| Jamal *et al.* 2016 | Wrong design: systematic review |
| Machi *et al.* 2009 | Wrong design: case series |
| Madsen *et al.* 1995 | Wrong publication type/study design: descriptive analysis of technique/instruments |
| Merhar *et al.* 1998 | Too few patients for comparison |
| Mosnier *et al.* 1992 | Wrong P: cholecystectomy, not laparoscopic |
| Noble *et al.* 2011 | Wrong I: choledochoscopy before LUS |
| Olsen *et al.* 1999 | Too few patients for comparison |
| Paolucci *et al.* 1995 | Wrong publication type/study design: descriptive analysis of technique/instruments |
| Pereira *et al.* 2020 | Wrong I: no laparoscopic ultrasonography |
| Piccolboni *et al.* 2008 | No comparison of I and C |
| Santambrogio *et al.* 1997 | No comparison of I and C |
| Shabanzadeh *et al.* 2022 | Wrong design: guidelines |
| Smulders *et al.* 1995 | Wrong publication type/study design: descriptive analysis of technique/instruments |
| Sun *et al.* 2016 | Wrong design: health economics |
| van de Graaf *et al.* 2018 | Wrong design: systematic review |
| Wu *et al.* 1998 | Too few patients for comparison |
| Zha *et al.* 2010 | I/C missing: descriptive analysis of 13,000 laparoscopic cholecystectomies |

C = comparison, I = intervention, P = Population, I; Intervention, LUS = laparoscopic ultrasonography, IOC = intraoperative cholangiography

**Table S2** Aspects regarding directness and study limitations identified during the assessment process contributing to the study being categorised as having no/minor (+), some (?) or major (-) problems. These assessments applied to all outcomes if not explicitly stated otherwise.

(For full citations, see p 8)

|  |  | **Problems contributing to downgrading the study in the assessment** | | | |
| --- | --- | --- | --- | --- | --- |
|  | **Study design** | **Directness** | | **Study limitations** | |
| Barteau *et al.* 1995 | Cross-sectional (intra-individual) | ? | Number of surgeons performing LUS not reported. Indication for surgery not reported (consecutive inclusion). | ? | Potential detection bias because of not blinded investigator, and LUS always performed prior to IOC. |
| Birth *et al.* 1998 | Cross-sectional (intra-individual) | ? | Only elective cholecystitis (consecutive inclusion). Number of surgeons performing LUS not reported. | + | Random order of LUS/IOC |
| Catheline *et al.* 1999 | Cross-sectional (intra-individual) | + |  | ? | Potential detection bias because of blinded investigator not reported, and LUS always performed prior to IOC. |
| Catheline *et al.* 2002 | Cross-sectional (intra-individual) | + |  | + |  |
| Halpin *et al.* 2002 | Before/after | ? | Only one surgeon performing LUS (consecutive inclusion) | ? | Some imbalances between groups in patient characteristics. Prevalence of acute cholecystitis not specifically reported. |
| Hublet *et al.* 2009 | Cross-sectional (inter-individual comparison) | ? | Only one surgeon performing LUS (consecutive inclusion) | - | Patient characteristics in comparison groups not reported. Not blinded assessments. |
| Li *et al.* 2009 | Cross-sectional (intra-individual) | - | Number of surgeons performing LUS not reported. Consecutive inclusion not described. Subgroup: inclusion according to score system (score 3 to 5, out of 9, included) | - | Numbers of procedural failure not clearly presented. Patients with iodine allergy and pregnancy excluded.  Not problematic for Imaging time |
| Machi *et al.* 1999 | Cross-sectional (intra-individual) | ? | Number of surgeons performing LUS not reported. Consecutive inclusion. | ? | Potential detection bias because of blinded investigator not reported, and LUS always performed prior to IOC. |
| Perry *et al.* 2008 | Cross-sectional (inter/intra-individual comparisons) | ? | Only one surgeon performing LUS (consecutive inclusion) | -/? | Patient characteristics in comparison groups not reported. Not blinded assessments. For the outcome diagnostic failure, these aspects were assessed as less problematic |
| Röthlin 1996 (Intra...) | Cross-sectional (intra-individual) | ? | Number of surgeons performing LUS not reported. Consecutive inclusion not described. | ? | Potential detection bias because of blinded investigator not reported, and LUS always performed prior to IOC. Patients (not all) included in Rothlin 1996b. |
| Röthlin *et al.*, 1996 (Laparo...) | Cross-sectional (intra-individual) | ? | Only one surgeon performing LUS (consecutive inclusion) | ? | Potential detection bias because of blinded investigator not reported, and LUS always performed prior to IOC. |
| Siperstein *et al.* 1999 | Cross-sectional (intra-individual) | ? | Number of surgeons performing LUS not reported. Characteristics of patients not reported. Consecutive inclusion. | ? | Potential detection bias because of blinded investigator not reported, and LUS always performed prior to IOC. |
| Stiegmann *et al.* 1995 | Cross-sectional (intra-individual) | + |  | ? | Potential detection bias because of blinded investigator not reported, and LUS always performed prior to IOC. |
| Thompson *et al.* 1998 | Cross-sectional (intra-individual) | ? | Only one surgeon performing LUS (consecutive inclusion) | ? | Potential detection bias because of blinded investigator not reported, and LUS always performed prior to IOC. |
| Tranter *et al.* 2003 | Cross-sectional (intra-individual) | ? | Only one surgeon performing LUS. Consecutive inclusion not described | ? | Potential detection bias because of blinded investigator not reported, and LUS always performed prior to IOC. |
| Wu *et al.* 1998 | Before/after | ? | Only one surgeon performing LUS (consecutive inclusion) | ?/+ | Some imbalances between groups in patient characteristics. One surgeon: before/after comparison |

LUS = laparoscopic ultrasonography, NA = not applicable, IOC = intraoperative cholangiography

**Full references**

Included studies

1. Barteau JA, Castro D, Arregui ME, Tetik C. A comparison of intraoperative ultrasound versus cholangiography in the evaluation of the common bile duct during laparoscopic cholecystectomy. Surg Endosc. 1995;9(5):490-6. doi: 10.1007/bf00206833.
2. Birth M, Ehlers KU, Delinikolas K, Weiser HF. Prospective randomized comparison of laparoscopic ultrasonography using a flexible-tip ultrasound probe and intraoperative dynamic cholangiography during laparoscopic cholecystectomy. Surg Endosc. 1998;12(1):30-6. doi: 10.1007/s004649900587.
3. Catheline J, Rizk N, Champault G. A comparison of laparoscopic ultrasound versus cholangiography in the evaluation of the biliary tree during laparoscopic cholecystectomy. Eur J Ultrasound. 1999;10(1):1-9. doi: 10.1016/s0929-8266(99)00028-2.
4. Catheline JM, Turner R, Paries J. Laparoscopic ultrasonography is a complement to cholangiography for the detection of choledocholithiasis at laparoscopic cholecystectomy. Br J Surg. 2002;89(10):1235-9. doi: 10.1046/j.1365-2168.2002.02198.x.
5. Halpin VJ, Dunnegan D, Soper NJ. Laparoscopic intracorporeal ultrasound versus fluoroscopic intraoperative cholangiography: after the learning curve. Surg Endosc. 2002;16(2):336-41. doi: 10.1007/s00464-001-8325-1.
6. Hublet A, Dili A, Lemaire J, Mansvelt B, Molle G, Bertrand C. Laparoscopic ultrasonography as a good alternative to intraoperative cholangiography (IOC) during laparoscopic cholecystectomy: results of prospective study. Acta Chir Belg. 2009;109(3):312-6. doi: 10.1080/00015458.2009.11680431.
7. Li JW, Feng B, Wu L, Wang ML, Lu AG, Zang L, et al. Intraoperative cholangiography in combination with laparoscopic ultrasonography for the detection of occult choledocholithiasis. Med Sci Monit. 2009;15(9):Mt126-30. doi.
8. Machi J, Tateishi T, Oishi AJ, Furumoto NL, Oishi RH, Uchida S, et al. Laparoscopic ultrasonography versus operative cholangiography during laparoscopic cholecystectomy: review of the literature and a comparison with open intraoperative ultrasonography. J Am Coll Surg. 1999;188(4):360-7. doi: 10.1016/s1072-7515(98)00313-5.
9. Perry KA, Myers JA, Deziel DJ. Laparoscopic ultrasound as the primary method for bile duct imaging during cholecystectomy. Surg Endosc. 2008;22(1):208-13. doi: 10.1007/s00464-007-9558-4.
10. Röthlin M. Intraoperative sonography during laparoscopic cholecystectomy. ProgSurg. 1996;22:144-9. doi.
11. Röthlin MA, Schöb O, Schlumpf R, Largiadèr F. Laparoscopic ultrasonography during cholecystectomy. Br J Surg. 1996;83(11):1512-6. doi: 10.1002/bjs.1800831107.
12. Siperstein A, Pearl J, Macho J, Hansen P, Gitomirsky A, Rogers S. Comparison of laparoscopic ultrasonography and fluorocholangiography in 300 patients undergoing laparoscopic cholecystectomy. Surg Endosc. 1999;13(2):113-7. doi: 10.1007/s004649900917.
13. Stiegmann GV, Soper NJ, Filipi CJ, McIntyre RC, Callery MP, Cordova JF. Laparoscopic ultrasonography as compared with static or dynamic cholangiography at laparoscopic cholecystectomy. A prospective multicenter trial. Surg Endosc. 1995;9(12):1269-73. doi: 10.1007/bf00190157.
14. Thompson DM, Arregui ME, Tetik C, Madden MT, Wegener M. A comparison of laparoscopic ultrasound with digital fluorocholangiography for detecting choledocholithiasis during laparoscopic cholecystectomy. Surg Endosc. 1998;12(7):929-32. doi: 10.1007/s004649900749.
15. Tranter SE, Thompson MH. A prospective single-blinded controlled study comparing laparoscopic ultrasound of the common bile duct with operative cholangiography. Surg Endosc. 2003;17(2):216-9. doi: 10.1007/s00464-002-8911-x.
16. Wu JS, Dunnegan DL, Soper NJ. The utility of intracorporeal ultrasonography for screening of the bile duct during laparoscopic cholecystectomy. J Gastrointest Surg. 1998;2(1):50-60. doi: 10.1016/s1091-255x(98)80103-0.

Excluded studies

1. Antal A, Janaki H. New diagnostic adjunct in laparoscopic procedures. Intraoperative ultrasound. Acta Chir Hung. 1994;34(1-2):151-60.
2. Awan B, Elsaigh M, Marzouk M, Sohail A, Elkomos BE, Asqalan A, et al. A Systematic Review of Laparoscopic Ultrasonography During Laparoscopic Cholecystectomy. Cureus. 2023;15(12):e51192. doi: 10.7759/cureus.51192
3. Aziz O, Ashrafian H, Jones C, Harling L, Kumar S, Garas G, et al. Laparoscopic ultrasonography versus intra-operative cholangiogram for the detection of common bile duct stones during laparoscopic cholecystectomy: a meta-analysis of diagnostic accuracy. Int J Surg. 2014;12(7):712-9
4. Bezzi M, Silecchia G, De Leo A, Carbone I, Pepino D, Rossi P. Laparoscopic and intraoperative ultrasound. Eur J Radiol. 1998;27:S207-S14. doi: 10.1016/S0720-048X(98)00064-3.
5. Biffl WL, Moore EE, Offner PJ, Franciose RJ, Burch JM. Routine intraoperative laparoscopic ultrasonography with selective cholangiography reduces bile duct complications during laparoscopic cholecystectomy. J Am Coll Surg. 2001;193(3):272-80. doi: 10.1016/s1072-7515(01)00991-7.
6. Bush AES, Christopoulos P, Jones RM, Sinha S, Srinivas G, Andrews SN. Safety, quality and efficiency of intra-operative imaging for treatment decisions in patients with suspected choledocholithiasis without pre-operative magnetic resonance cholangiopancreatography. Surg Endosc. 2022;36(2):1206-14. doi: 10.1007/s00464-021-08389-y.
7. Catheline JM, Turner R, Rizk N, Barrat C, Buenos P, Champault G. Evaluation of the biliary tree during laparoscopic cholecystectomy: laparoscopic ultrasound versus intraoperative cholangiography: a prospective study of 150 cases. Surg Laparosc Endosc. 1998;8(2):85-91.
8. Chandra A, Gupta V, Rahul R, Kumar M, Maurya A. Intraoperative ultrasonography of the biliary tract using saline as a contrast agent: a fast and accurate technique to identify complex biliary anatomy. Can J Surg. 2017;60(5):316-22. doi: 10.1503/cjs.011116.
9. Deziel DJ. Laparoscopic Ultrasound for Bile Duct Imaging during Cholecystectomy: Clinical Impact in 785 Consecutive Cases. J Am Coll Surg. 2022;234(5):849-60. doi: 10.1097/xcs.0000000000000111.
10. Dili A, Bertrand C. Laparoscopic ultrasonography as an alternative to intraoperative cholangiography during laparoscopic cholecystectomy. World J Gastroenterol. 2017;23(29):5438-50. doi: 10.3748/wjg.v23.i29.5438.
11. Fisher AT, Bessoff KE, Khan RI, Touponse GC, Yu MMK, Patil AA, et al. Evidence-based surgery for laparoscopic cholecystectomy. Surg Open Sci. 2022;10:116-34. doi: 10.1016/j.sopen.2022.08.003.
12. Hashimoto M, Matsuda M, Watanabe G. Intraoperative ultrasonography for reducing bile duct injury during laparoscopic cholecystectomy. Hepatogastroenterology. 2010;57(101):706-9.
13. Jakimowicz J. Intraoperative and Postoperative Biliary Endoscopy - Intraoperative Ultrasonography and Sonography during Laparoscopic Cholecystectomy. Problems in General Surgery. 1991;8(3):442-57.
14. Jakimowicz JJ. Review: intraoperative ultrasonography during minimal access surgery. J R Coll Surg Edinb. 1993;38(4):231-8.
15. Jamal KN, Smith H, Ratnasingham K, Siddiqui MR, McLachlan G, Belgaumkar AP. Meta-analysis of the diagnostic accuracy of laparoscopic ultrasonography and intraoperative cholangiography in detection of common bile duct stones. Ann R Coll Surg Engl. 2016;98(4):244-9. doi: 10.1308/rcsann.2016.0068.
16. Machi J, Johnson JO, Deziel DJ, Soper NJ, Berber E, Siperstein A, et al. The routine use of laparoscopic ultrasound decreases bile duct injury: a multicenter study. Surg Endosc. 2009;23(2):384-8. doi: 10.1007/s00464-008-9985-x.
17. Madsen MR, Mortensen PM, Hovendal CP. Laparoscopic ultrasonography. A review and authors' own experiences. Ugeskr Laeger. 1995;157(5):575-80.
18. Merhar GL, Shelley DJ, Fegelman E. Laparoscopic ultrasound invades the abdomen. Diagn Imaging (San Franc). 1998;Suppl:Au21-5.
19. Mosnier H, Audy JC, Boche O, Guivarc'h M. Intraoperative sonography during cholecystectomy for gallstones. Surg Gynecol Obstet. 1992;174(6):469-73.
20. Noble H, Norton S, Thompson M. Assuring complete laparoscopic clearance of the bile duct. J Laparoendosc Adv Surg Tech A. 2011;21(4):319-22. doi: 10.1089/lap.2010.0433.
21. Olsen AK, Bjerkeset OA. Laparoscopic ultrasound (LUS) in gastrointestinal surgery. Eur J Ultrasound. 1999;10(2-3):159-70. doi: 10.1016/s0929-8266(99)00053-1.
22. Paolucci V, Schaeff B, Encke A. The role of laparoscopic ultrasonography of the biliary tree. Problems in General Surgery. 1995;12(3):35-45.
23. Pereira J, Bass GA, Mariani D, Dumbrava BD, Casamassima A, da Silva AR, et al. Surgeon-performed point-of-care ultrasound for acute cholecystitis: indications and limitations: a European Society for Trauma and Emergency Surgery (ESTES) consensus statement. Eur J Trauma Emerg Surg. 2020;46(1):173-83. doi: 10.1007/s00068-019-01197-z.
24. Piccolboni D, Ciccone F, Settembre A, Corcione F. The role of echo-laparoscopy in abdominal surgery: five years' experience in a dedicated center. Surg Endosc. 2008;22(1):112-7. doi: 10.1007/s00464-007-9382-x.
25. Santambrogio R, Montorsi M, Bianchi P, Opocher E, Verga M, Panzera M, et al. Common bile duct exploration and laparoscopic cholecystectomy: role of intraoperative ultrasonography. J Am Coll Surg. 1997;185(1):40-8. doi: 10.1016/s1072-7515(97)00013-6.
26. Shabanzadeh DM, Christensen DW, Ewertsen C, Friis-Andersen H, Helgstrand F, Nannestad Jørgensen L, et al. National clinical practice guidelines for the treatment of symptomatic gallstone disease: 2021 recommendations from the Danish Surgical Society. Scand J Surg. 2022;111(3):11-30. doi: 10.1177/14574969221111027.
27. Smulders JF, Jakimowicz JJ. Color Doppler application in laparoscopic intraoperative ultrasonography. Surg Technol Int. 1995;Iv:183-7.
28. Sun SX, Kulaylat AN, Hollenbeak CS, Soybel DI. Cost-effective Decisions in Detecting Silent Common Bile Duct Gallstones During Laparoscopic Cholecystectomy. Ann Surg. 2016;263(6):1164-72. doi: 10.1097/sla.0000000000001348.
29. van de Graaf FW, Zaïmi I, Stassen LPS, Lange JF. Safe laparoscopic cholecystectomy: A systematic review of bile duct injury prevention. Int J Surg. 2018;60:164-72. doi: 10.1016/j.ijsu.2018.11.006.
30. Wu JS, Dunnegan DL, Luttmann DR, Soper NJ. The evolution and maturation of laparoscopic cholecystectomy in an academic practice. J Am Coll Surg. 1998;186(5):554-60; discussion 60-1. doi: 10.1016/s1072-7515(98)00052-0.
31. Zha Y, Chen XR, Luo D, Jin Y. The prevention of major bile duct injures in laparoscopic cholecystectomy: the experience with 13,000 patients in a single center. Surg Laparosc Endosc Percutan Tech. 2010;20(6):378-83. doi: 10.1097/SLE.0b013e3182008efb.
